# Neck pain in episodic migraine: premonitory symptom or part of the attack?

**DOI:** 10.1186/s10194-015-0566-9

**Published:** 2015-09-02

**Authors:** Christian Lampl, Mirjam Rudolph, Christina I. Deligianni, Dimos D. Mitsikostas

**Affiliations:** Medical Headache Center, Hospital Sisters of Mercy, Seilerstaette Linz, Linz, 4020 Austria; Department of Neurology, Athens Naval Hospital, Athens, 11521 Greece

## Abstract

**Background:**

Whether neck pain (NP) is a prodromal migraine symptom or belongs to the migraine attack feature remains controversial.

**Methods:**

In order to prospectively record neck pain (NP) and non-headache symptoms and to evaluate the percentage of patients having NP as clear premonitory, non-headache symptom of their migraine, a specific self fulfilled questionnaire was designed to record NP and premonitory symptoms in a migraine cohort. All patients who reported NP anytime during the migraine phase were allocated to 3 groups: A = NP starts with the onset of headache; B = NP starts < 2 h before the onset of headache; C = NP starts 2-48 h before the onset of headache.

**Results:**

Data were evaluated from 487 migraineurs with episodic migraine (73.1 % females; 77 % had migraine without aura). 338 patients (69.4 %) reported NP anytime during the migraine phase. 184 patients (group A; 54.4 %) noticed NP with the start of the headache phase; 118 patients (group B; 24.2 %) reported NP within 2 h before the headache phase; 36 patients (group C; 7.4 %) experienced NP 2-48 h before the headache phase. In group B we found a high proportion of typical migraine associated symptoms and NP progressed into the headache phase in 82.2 %.

**Conclusions:**

These data indicate that NP is a very common feature of migraine attacks and is more likely to be part of the migraine attack than a prodromal migraine symptom.

## Background

Neck pain (NP) ranks among the most common complaints in medicine, affecting 14–71 % of adults [1]. Migraine is a disorder affecting 10–15 % of people worldwide [2]. In adults, different features of NP such as pericranial muscle tenderness, myofascial referred pain from neck muscles, and the dysfunction of the joints of the upper cervical spine have been associated with headache [3, 4]. Hence, the overlap in clinical features between NP and migraine has added to the controversy. Many migraine sufferers report neck discomfort and stiffness before and/or during an attack. Although the pain of migraine is most commonly perceived in the ophthalmic distribution of the trigeminal nerve, a substantial percentage of migraineurs reported to experience pain in the neck and occiput with their attacks [5]. In one of first studies in the early 90 it was reported that one third of patients have NP in the prodromal (premonitory) state, again one third in the postdromal phase of a migraine neck [6]. In a recently published population study the 1 year prevalence of NP was 68.4 % and higher in those with vs. without primary headache, 76 % of pure migraine sufferers reported NP [7].

In general medicine a prodrome is an early symptom (or set of symptoms) that might indicate the start of a disease before specific symptoms occur. Prodromes may be non-specific symptoms or, in a few instances, may clearly indicate a particular disease, such as the prodromal migraine aura. Hence, the revised IHS classification ICHD-III beta [8] provided further notes to better clarify the distinction. In the “definition of terms” section, prodromal symptoms are defined as “symptoms preceding and forewarning of a migraine attack by 2–48 h, occurring before the aura in migraine with aura and before the onset of pain in migraine without aura. Prodromal symptoms occur in ~ 60 % of those with migraines with an onset of 2–48 h before the start of pain, typical migraine features or the aura [9].

The question we would like to answer is, if NP can clearly be premonitory symptom or, moreover, is NP part of the migraine attack? To answer this question we designed a questionnaire, which was validated in two different tertiary headache centres.

The objectives of this study were twofold: first, to prospectively record NP and non-headache symptoms from a cohort of migraineurs and secondly, to evaluate the percentage of patients having NP as clear prodromal symptom of their migraine.

## Methods

NP is defined as a subjective unpleasant sensory experience in the neck. It may be manifested as radiating to the upper extremities or the head, according to the definition of Merskey and Bogduk [10]. In our questionnaire NP as a prodromal (premonitory) symptom was defined as NP (uni- or bilateral) that occurs 2–48 h before the aura in migraine with aura and before the onset of head pain in migraine without aura (according to the ICDH-III beta) and without any additional accompanying symptoms (sensitivity to light/sound/smell, vertigo, nausea, vomiting, aggravating pain by physical activity e.g. climbing stairs, walking etc.). If NP occurred in a time period of 2 h before the aura in migraine with aura and before the onset of pain in migraine without aura, with any accompanying typical migraine symptoms it was interpreted as part of the migraine attack itself.

Inclusion criteria are: both sexes aged 18–65 year, episodic migraine with and/or without aura pre-diagnosed by a neurologist according to the definition of the second edition of the International Classification of Headache Disorders [8], ability to distinguish NP and migraine from other interval headaches. Patients with known or suspected cervicogenic headache, history of significant cervical trauma or surgery, fibromyalgia and any general pain syndrome were excluded.

For this evaluation an online questionnaire was set on the homepages of the Austrian self-helping group (www.shgkopfweh.at) and the headache Medical Center Seilerstaette Linz (www.kopfschmerz-linz.at). Further identical questionnaires were delivered in identical envelopes in the waiting room of the Headache Medical Center Seilerstaette Linz. Besides a written explanation of NP and the different migraine phases (prodromal, aura, headache, postdromal), the questionnaire consists of demographic data, questions about the inclusion and exclusion criteria, if the individual migraine was pre-diagnosed by a neurologist and the following questions to be answered for three consecutive attacks:

Did you experience NP anytime during the headache phase?

Did you experience NP with the onset of headache?

Did you experience NP < 2 h before the onset of headache?

Did you experience NP 2–48 h before the onset of headache?

For all these questions we additionally asked for the side and quality of NP (stabbing, cramping, lancinating, pulsating, numb). Associated migraine symptoms like sensitivity to light, sound and smell, nausea, vomiting, vertigo, aggravation of pain by physical activity (e.g. climbing stairs, walking etc.) were also recorded. For the last two questions we also asked if NP progressed into the headache phase.

The study conformed to the revised ethical principles of the Helsinki declaration and the Codex rules and guidelines for research [11, 12].

All patients who reported neck any time during the migraine phase were allocated to 3 groups: A = NP starts with the onset of headache; B = NP starts < 2 h before the onset of headache; C = NP starts 2–48 h before the onset of headache. All questions ideally should be answered immediately after the next migraine attack, but no longer than 6 h after the end of the attack. Data were summarized using descriptive statistics: either number or percentage of patients in each category or number of patients, mean, SD, median, and range.

## Results

**Fig. 1:**
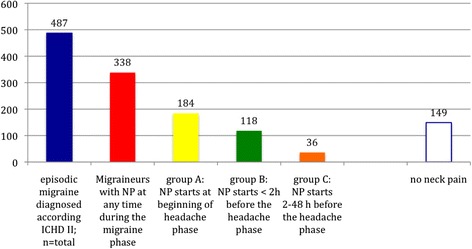
Numbers of migraineurs per group with neck pain over the past 3 migraine attacks in the total study population (*n* = 487; 356 females [73.1 %] and 131 male [26.9 %])

Five hundred twelve patients returned the questionnaire. 25 patients had to be excluded for further evaluation because Q1 was answered with “no” or not answered at all. Demographic details: the study consists of 487 migraineurs with episodic migraine (Fig. 1; 356 females [73.1 %] and 131 male [26.9 %]). 77 % of patients had migraine without aura; mean age was 38 years (range, 19 to 61 years); mean age at onset of migraine was 21 years (range, 11 to 42 years). The median number of attacks during the study period was 6 per month (range 3–14).

**Fig. 2:**
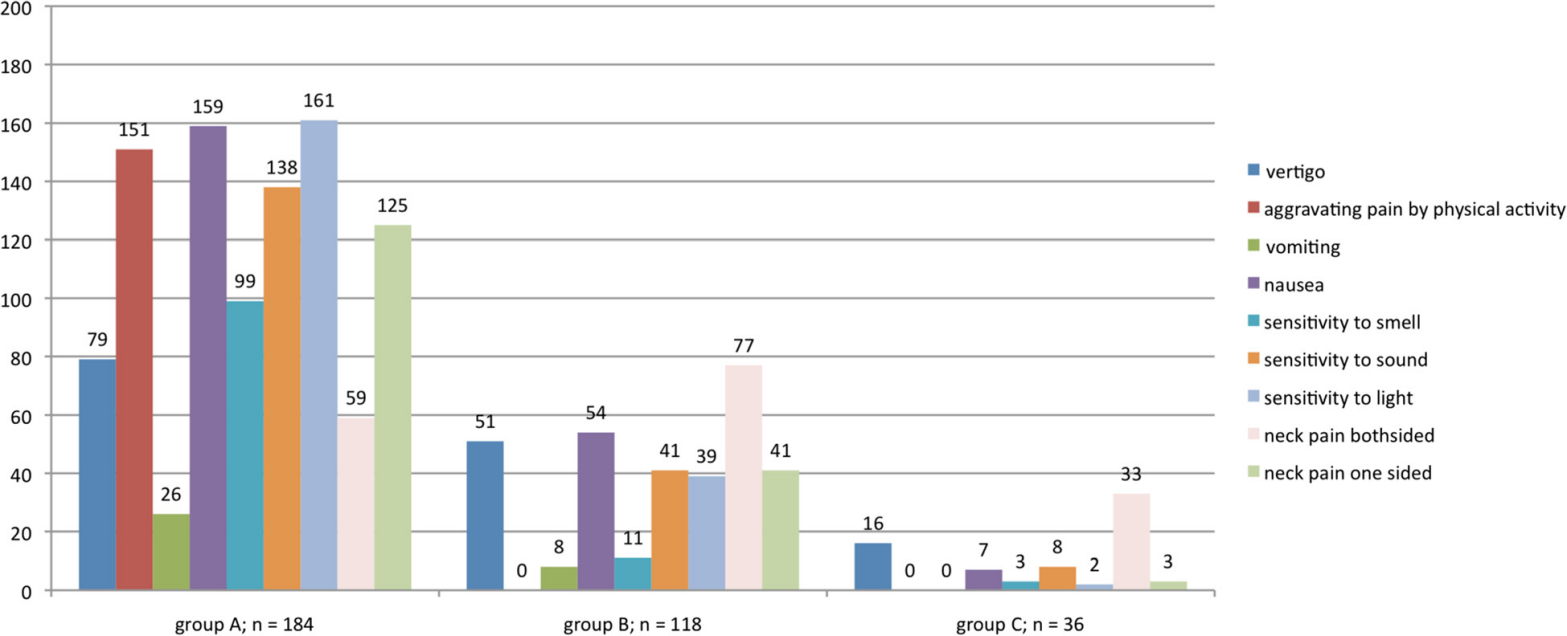
Migraine associated symptoms and NP in different groups

Three hundred thirty eight patients (69.4 %) reported NP anytime during the migraine phase. 184 patients (group A; 54.4 %) noticed NP with the start of the headache phase (bilateral: *n* = 59; 32.1 %; unilateral: *n* = 125; 67.9 %); 118 patients (group B; 24.2 %) reported NP within 2 h before the headache phase (bilateral: *n* = 41; 34.7 %; unilateral: *n* = 77; 65.2 %) and progressed into the headache phase in 97 patients (82.2 %); 36 patients (group C; 7.4 %) experienced NP 2–48 h before the headache phase (bilateral: *n* = 3; 8.3 %; unilateral: *n* = 33; 91.7 %), and progressed into the headache phase in 8 patients (22.2 %). Fig. 2 shows all patients having NP at different time points with their specific associated symptoms.

In group A all patients showed typical migraine related symptoms: 42.9 % experienced vertigo, 82 % had an aggravation of pain during physical activity, 86.4 % had nausea, 53.8 % were sensitive to smell, 75 % showed sensitiveness to sound and 87.5 % to light. In group B 43.2 % experienced vertigo, 45.8 % had nausea, 9.3 % were sensitive to smell, 34.7 % showed sensitiveness to sound and 33 % to light. In group C 44.4 % reported vertigo, 8.3 % hypersensitivity to smell, 22.2 % to sound and 5.5 % to light. Quality of NP was crampy (81,7 %), throbbing (53.0 %), stabbing (47,5 %), numb (24,8 %). We found no association between patients reporting migraine with and without aura.

## Discussion

The present results are based on a cohort of patients with episodic migraine, pre-diagnosed through neurologists. The high participation rate indicates that the study cohort represents a migraine population without any serious selection bias. We found that in 69 % of migraine patients NP is present anytime during a migraine phase. This result is in line with a recently published article of the Danish Headache group [7]. In our population study 54 % noticed NP with the start of the headache phase, in 24 % NP occurred in a time period of 2 h before the aura in migraine with aura and before the onset of pain in migraine without aura. Of interest is the high percentage of vertigo (43 %), nausea (46 %), sensitive to sound (35 %) and light (33 %) in this group. Vertigo, nausea, sensitivity to sound and light are typical symptoms of migraine, so that we can hypothesize that in that particular group NP is also part of the migraine itself (in 82 % NP progressed into the headache phase) and not (as per definition) a prodromal symptom. Giffin et al. [13] reported similar high percentages begging the question of when the premonitory phase does end and the headache phase begins? They consider that the headache evolves from the premonitory phase over a variable period, with the full-blown migraine headache finally developing when a critical physiologic threshold is reached. In a multicenter, electronic diary study, that evaluates, if premonitory symptoms can accurately predict the full-blown headache, NP (“stiff neck”) was seen premonitory in 50 % of patients, during headache in 63 % [13]. 7.4 % of our patients experienced NP in the prodromal phase (as per definition 2–48 h before the headache phase). However, in this group we also found a high proportion of vertigo (44 %), phonophobia (22 %) and photophobia (6 %).

Prodromal (premonitory) symptoms can widely range from patient to patient or even from headache to headache within the same patient. These symptoms often begin hours to days before the onset of the headache phase of migraine. Blau reported that many patients have a period early in the migraine process of “headache awareness” where the headache has yet to declare its path of evolution [6]. Consequently, patients may delay therapy as they “wait and see” which presentation of the migraine process will unfold. This prompts patients to postpone treatment or to employ treatment beyond the point when it is likely to be effective. Patients then may experience unnecessary disability and unwarranted risk from utilizing medications when they are unlikely to provide therapeutic value.

The pathophysiology of the prodrome is largely unstudied. The varied nature of symptoms associated with prodrome, however, suggests that central disruption occurs at both cortical and subcortical levels. The association of NP and migraine may result from various pathophysiological mechanisms. No studies have been carried out of the possible causal association of NP and migraine, neither is it known whether a reciprocal association occurs between NP and migraine. The structures of the neck innervated by the first three cervical nerves can be associated with migraine through the convergence of nociceptive afferents at the level of the caudal part of the trigeminal nucleus in the brainstem and sensitization of trigeminocervical neurones [14–17]. According to the first current hypothesis, NP has been regarded as a peripheral reflexion of migraine. Very often people report that their migraine “starts” in the neck and they implicate that their neck is the “cause” of their migraine. Prolonged nociceptive stimuli from the neck structures could also be important for producing continuous afferent bombardment of the trigeminal nerve nucleus caudalis, and, hence, activation of the trigeminovascular system [18–20]. As the pathogenesis of migraine is linked to the trigeminal innervations of the cranial blood vessels, noxious stimuli from the cervical structures may also play a role in this pathogenesis by facilitating central sensitization [21].

The advantage of the present study is a large sample of well-characterized individuals with migraine. We tried to find a simple way of measuring NP in adolescent HA sufferers with only a few questions.

Our study has several limitations: As in most previous epidemiological studies in headache, we did not use a daily headache diary. The classification of migraine was not based on a structured interview. We were not able to control if all patients included in the analysis had a pre-diagnosed migraine throughout a neurologist according to the IHS criteria. If migraine patients have different kinds of headache (although they were excluded), they probably recall the most frequent one when filled out the questionnaire, and the nature of other types, especially tension-type headache and medication-overuse headache remains obscure without a headache diary. As NP was determined throughout a simple questionnaire we were not able to perform physical examination and measurements in case of pericranial muscle tenderness and myofascial referred pain that may also contribute to migraine. Psychological factors such as anxiety and depression (in prodromal phase in particular), which have modulating effects on pain perception [22], cannot be ruled out in our cohort. As both pain syndromes, NP and migraine are regarded as due to a common risk factor, such as individual, physical, psychosocial, familial and genetic risk factors, these could predispose to general pain “proneness”. Further, the subject’s willingness to report symptoms cannot be ruled out as a contributing factor.

## Conclusions

In conclusions, NP has been found to be a common part of migraine episodes. This study, while not conclusive, support the concept for NP being related to the disorder itself, as in 78 % of patients, NP that starts with the headache or within 2 h before the headache and progresses into the headache phase of the migraine episode. NP could be a peripheral cause of migraine but also a manifestation of central mechanisms. This theory is supported by studies showing that nociceptive afferents from the meninges and the upper three cervical nerves from cervical structures converge to the same second-order neurons in the trigeminocervical complex. These considerations imply that it is an integral component of the migraine process that could have implications for treatment. To find appropriate interventions, we plan to perform a prospective, follow-up study with emphasis on different treatment time points (2–48 h before headache starts, < 2 h before headache starts, at headache start) and their impact on NP and migraine. It may be possible that there are differences in the treatment of migraine depending on the characteristics of co-occuring NP. Furthermore, prevention and treatment of NP could be important in the prevention of future chronic migraine.

## Consent

Written informed consent was obtained from the patient for the publication of this report and any accompanying images.
